# Motion preservation surgery: excision of juxta C5–C6 intervertebral disc osteoid osteoma using 3D C-arm based navigation: technical report

**DOI:** 10.1051/sicotj/2018052

**Published:** 2018-12-05

**Authors:** Arvind Kulkarni, Ankit Patel

**Affiliations:** 1 Mumbai Spine Scoliosis & Disc Replacement Centre, Bombay Hospital & Medical Research Center Marine Lines Mumbai 400002 India; 2 Saifee Hospital, Maharishi Karve Marg Charni Road Mumbai India

**Keywords:** Osteoid osteoma, 3D Navigation, Minimally invasive, Preserved motion segment, Cervical spine, Spine surgery, Excision.

## Abstract

*Introduction*: Precise targeted excision of the C5–C6 osteoid osteoma with placement of reference array on clavicle with minimal disturbance of anatomy and motion.

*Methods*: A 20-year-old male presented with an osteoid osteoma in the superior end plate of the C6 vertebra abutting the spinal canal causing intractable pain. The authors curetted the nidus using a 3D C-arm-based intraoperative scan integrated with an optical navigation system through a minimal access anterior cervical exposure. The patient reference array was affixed to the left clavicle using a threaded pin.

*Results*: The postoperative CT-scan revealed complete excision. Follow-up MRI and CT after 12 months revealed C5–C6 intervertebral disc to be intact without evidence of any tumor recurrence. VAS for neck pain improved from 8/10 to 2/10 immediately postoperatively and 0/10 at 1 year follow-up with no limitation of cervical movement. A motion segment was preserved with this technique.

*Conclusions*: Navigation allowed safe curettage of the nidus with minimal disturbance to the anatomy and motion. The site of attachment of patient reference array on clavicle can be recommended as stable, meeting all the criteria for optimal accuracy and stability.

## Introduction

Cervical spine accounts for 26.8% of all spinal osteoid osteomas. Surgical excision is recommended for patients unresponsive to anti-inflammatory drugs [1,2] . Intraoperative localization of the nidus, which can be extremely challenging, governs clinical results [3,4]. Surgical techniques of the spine have evolved, and minimally invasive procedures are now utilized to decompress the spinal cord with high precision. Posterior micro-foraminotomy, lamino-foraminotomy, cervical anterior/posterior endoscopic approaches and minimally invasive laminectomy are examples of such procedures [5]. A careful patient selection is essential in these cases. The authors report the first description of a technique of excision of an osteoid osteoma of the C6 body proximal to the spinal cord as well as the superior end plate using intraoperative navigation. Precise localization and excision of the juxta-intervertebral disc lesion helped in avoiding fusion and maintained motion of the C5–C6 segment. Clavicle served as the anchor for minimal invasively placed patient reference array (PRA). A novel application of the already described anterior trans-corporeal tunnel approach in treatment of cervical spondylotic myelopathy and herniated discs was utilized in the described case preserving segmental motion and function [6,7].

### Technical note/case report

**Figure 1:**
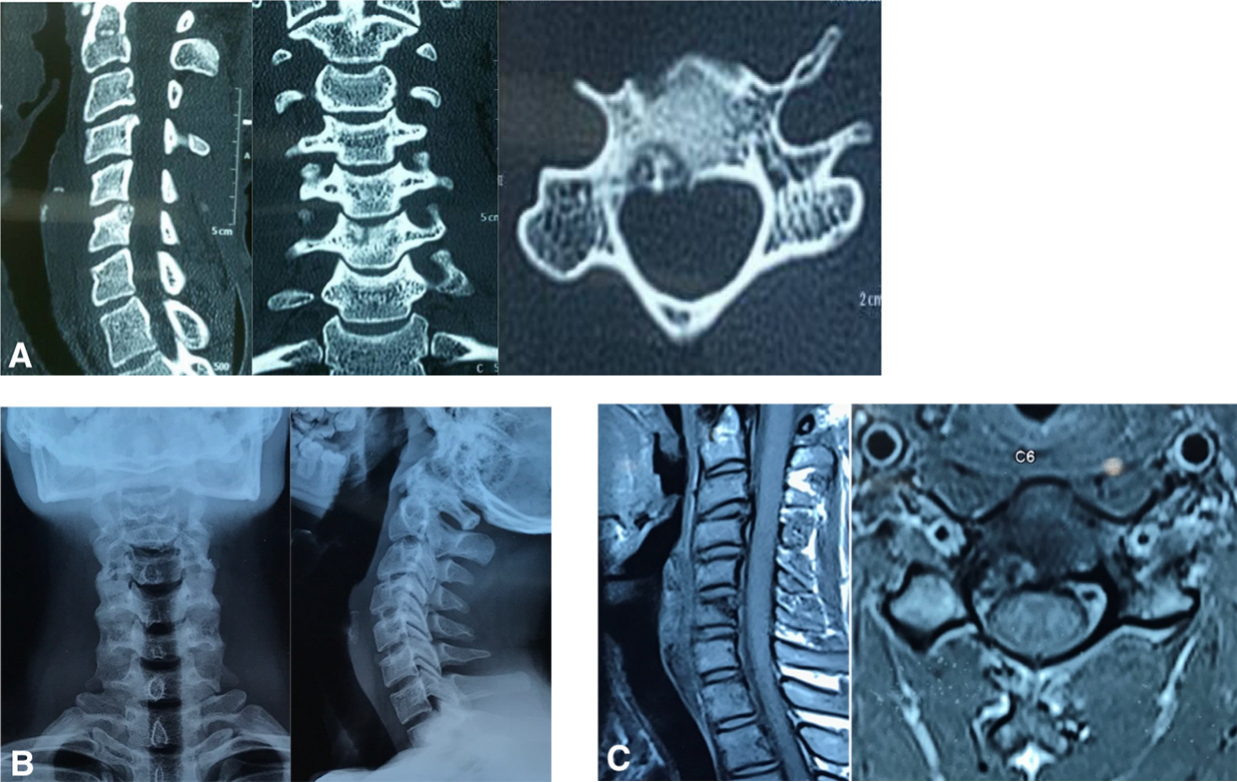
(a) Preoperative CT scan with the nidus in C6 vertebral body abutting the end plate and posterior wall. (b) Plain X-rays not depicting the lesion. (c) MRI T1 sagittal and axial images showing the lesion with peripheral edema.

**Figure 2:**
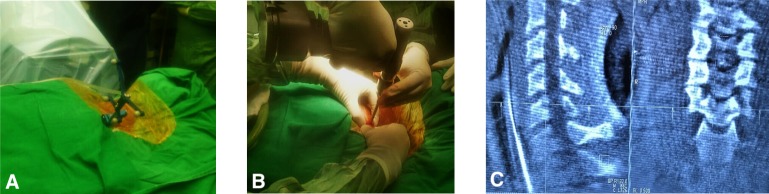
Intraoperative pictures. (a) Patient reference array attachment on left clavicle. (b) Drilling for threaded pin placement. (c) 3D CT image depicting the location of osteoid osteoma.

**Figure 3:**
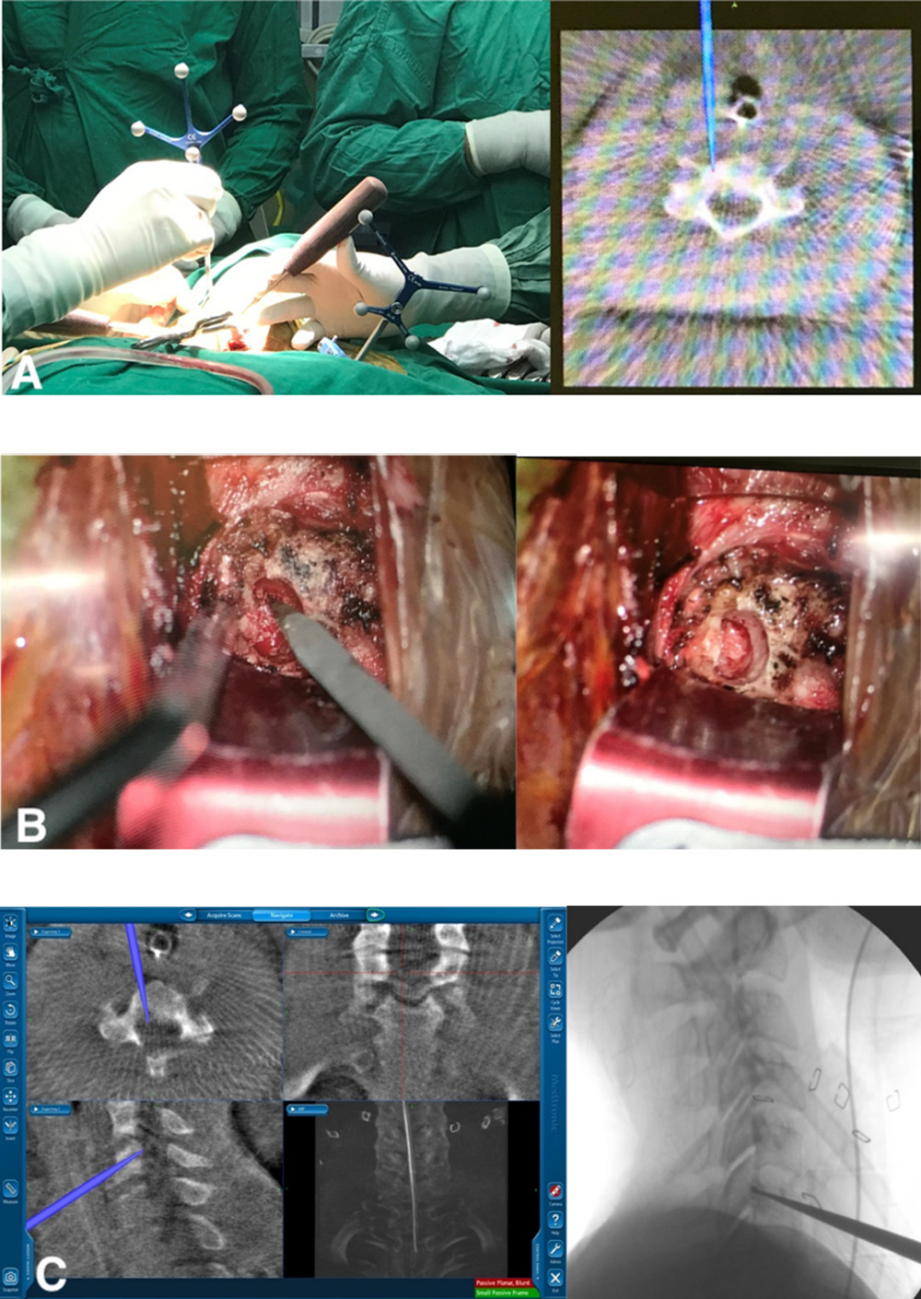
(a) Use of navigation for surface location. (b) Precise drilling toward the lesion using guided probe. (c) Checking finally adequate intralesional excision.

**Figure 4:**
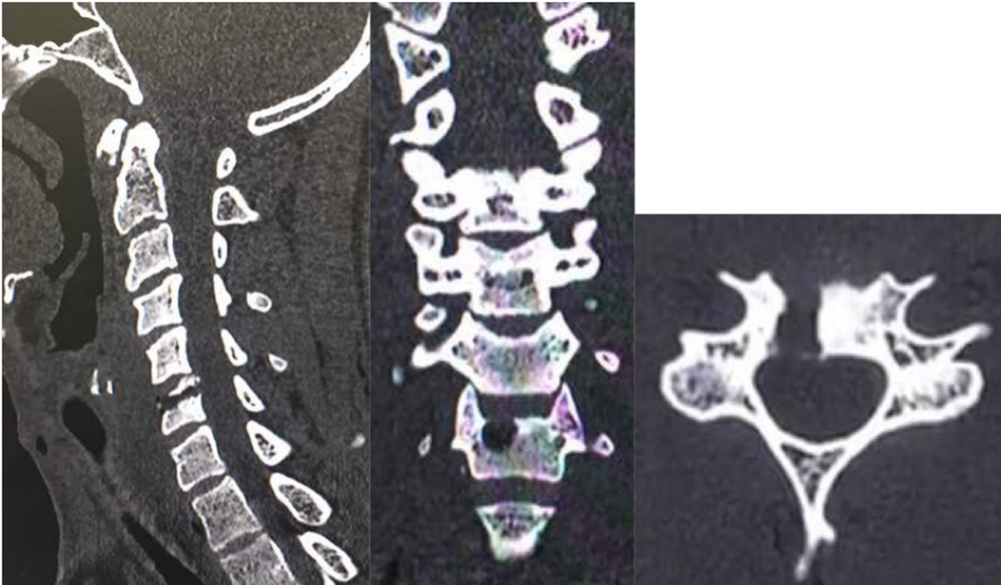
Postoperative CT scan demonstrating adequate excision with preservation of bony end plate.

**Figure 5:**
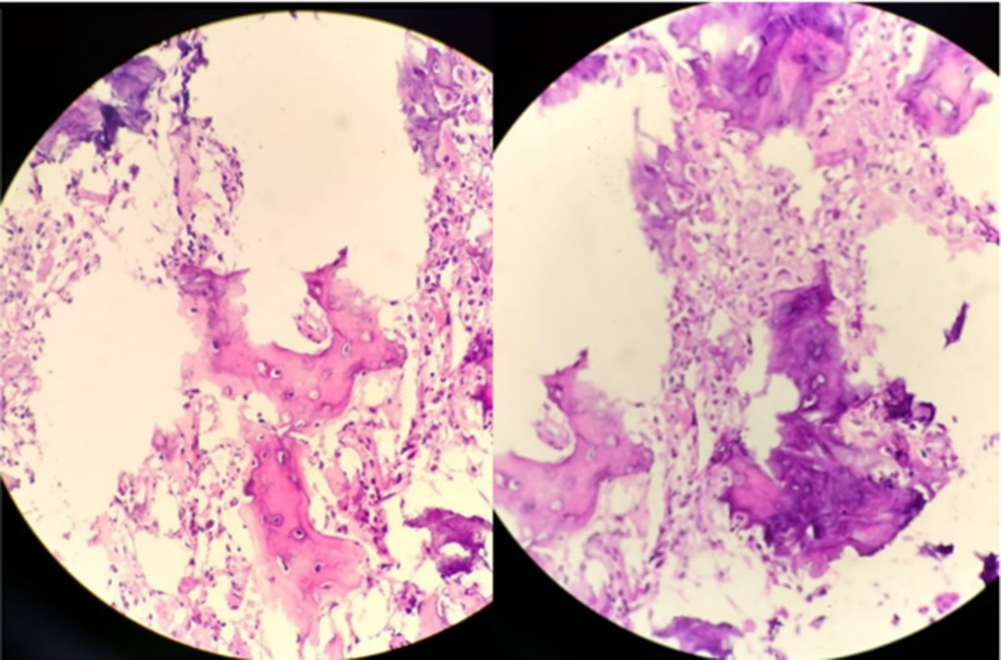
Photomicrographs at 400× magnification show osteoid trabeculae separated by fibrovascular tissue and lined by osteoblasts (stain: hematoxylin and eosin).

**Figure 6:**
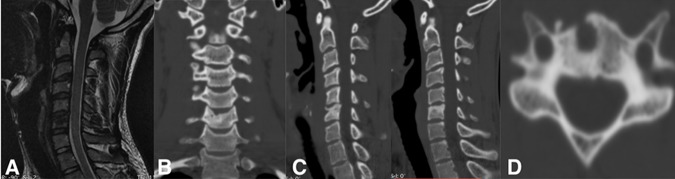
One-year follow-up imaging depicting (a) MRI sagittal view with preservation of adjacent disc; (b) CT scan coronal; (c) sagittal; and (d) axial views with filling of cavity with new bone formation and preservation of disc height and stability.

**Figure 7:**
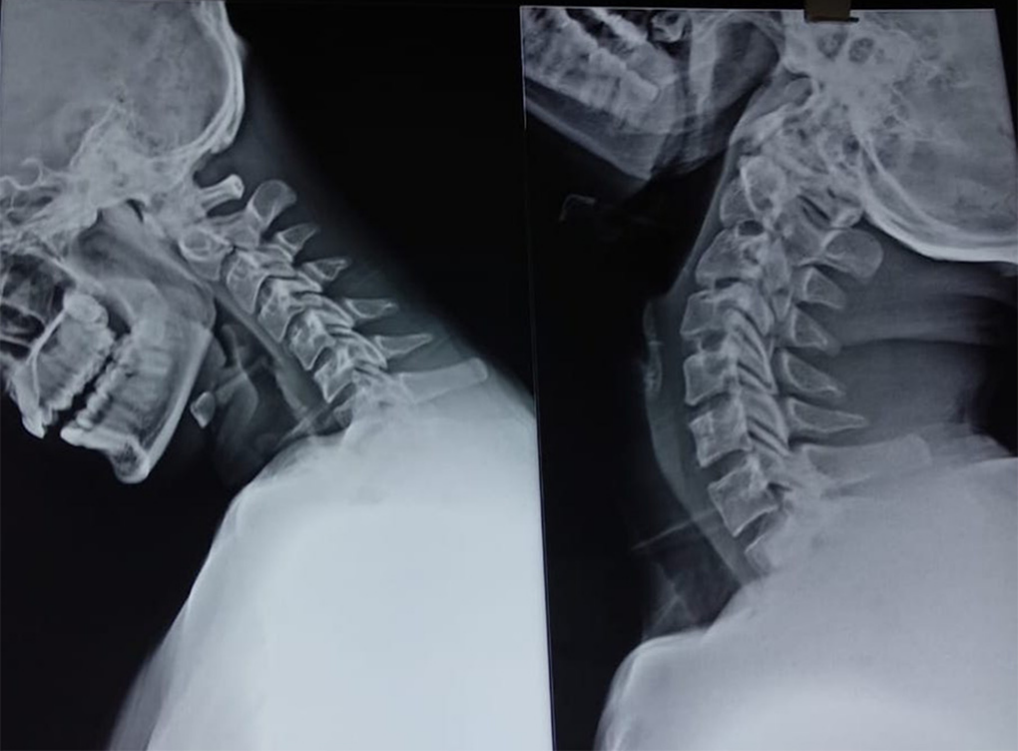
One-year follow-up flexion and extension X-rays show stability and preservation of the motion segment.

A 20-year-old male with a 2-year history of neck pain of progressively increasing severity had been resilient to NSAIDs. CT scan showed an oval osteolytic lesion with ossified nidus and surrounding sclerosis suggestive of an osteoid osteoma at the right posterosuperior corner of C6 vertebral body abutting the superior end plate and close to the spinal cord. The lesion measured 6.3 × 5.6 × 6 20 mm in dimensions (Figure 1). A plan of intraoperative 3D C-arm-based navigation was finalized (Stealth-Station S7, Medtronic; SIEMENS Arcadis Orbic 3D C-arm) for precise location and excision of the lesion with preservation of adjacent disc and cervical mobility (Figure 2). A prerequisite of successful 3D spin data set integration to the navigation workstation demands affixing the patient reference array (PRA) to an accessible fixed bony point closest to the region-of-interest which maximizes the accuracy of a navigable instrument while maintaining a free operational corridor. A decision to use middle-third of clavicle was taken. The head and shoulders were taped down to the operating table to prevent rotational movements of neck and indirect movement of clavicle along with shoulder. A right-sided approach was taken through a 2-cm horizontal skin-crease incision after external level marking using the navigated probe. Surface localization of the hidden osteoid osteoma was made and careful drilling initiated with the help of real-time navigation using the navigated probe. The probe guided in preserving the adjacent end plate and the disc to reach the ventral surface of the lesion. The cavity was curetted followed by drilling of walls for completion of intralesional extirpation (Figure 3). The patient was completely relieved of the pain. A postoperative CT scan done showed adequate excision with preservation of adjacent end plate and the intervertebral disc (Figure 4). Histopathology showed osteoid osteoma (Figure 5). Follow-up scans at 1 year show no local recurrence, a healthy C5–C6 intervertebral disc and CT scan depicting formation of bone at the site of excision (Figure 6). Follow-up flexion and extension X-rays at 1 year show stability and preservation of the motion segment (Figure 7).

## Discussion

Localization of the nidus is a challenge in osteoid osteoma. Intraoperative gamma probe is one such technique used in localization [8]. However, wide surgical excision and additional fusion may still be necessary since the lesion is usually not grossly visible [9–11]. CT-guided percutaneous localization and resection of nidus using a drill has been described for osteoid osteomas of the extremities and spine [12]. However, the results of this technique are questionable in the spine [8,3]. Again, percutaneous procedures were ruled out in this case in view of the related vital structures. Percutaneous radio-frequency thermal ablation and laser photocoagulation have been promoted as minimally invasive treatment options for spinal osteoid osteomas. However, thermal necrosis denying histologic verification of the specimens[13,14], risk of neurovascular injury [15], incomplete resection and recurrent symptoms necessitating reoperation are some of the problems with radio frequency ablation [16,17]. Intraoperative 3D navigation has been previously utilized for resection of osteoid osteomas and other bony lesions of extremities as well as spine [18–20]. The placement of PRA is crucial to the successful data integration and accuracy of real-time tracking of registered instruments. The stable foundation of PRA is commonly achieved by clamping on to the nearby spinous process or tri-flanged pin placement in iliac crest for lumbar spine procedures or a 3-point head-holder-based external array used in intracranial surgeries. To navigate in the anterior cervical spine, a detailed technique has not been described yet with the patient in supine position. Clavicle, being a subcutaneous bone with minimally mobile attachments at both ends and in close proximity to the anterior cervical spine, is ideally suitable for the purpose and hence can be routinely used [4]. The cortical tubular nature of the middle third of clavicle is technically more suitable for a minimally invasive threaded pin (Caspar distractor pin) placement and the resultant small scar gets concealed well under clothing. In this particular case, precise localization and excision of the precariously placed nidus using navigation in all three axes benefited the patient. Anterior transcorporeal tunnel approach has variably been used by different authors for patients with focal compression causing cervical spondylotic myelopathy or a disc herniation but its utilization in excision of a nidus of osteoid osteoma has not been described in literature [4,6]. C5–C6 segment being significantly mobile, especially in a young patient was not fused, thus saving motion. Avoiding fusion helped in reducing the invasiveness, morbidity, pain, costs, and all the repercussions of motion sacrifice. Use of microscope and navigation assisted in making sure that the surgery was safe with no neurovascular injury.

## Conclusion

The intraoperative 3D navigation system allowed a minimally invasive pin-point approach to localize and excise in real-time a precariously seated osteoid osteoma without causing any neurovascular injury and saving intervertebral motion with complete pain relief. Employment of minimally invasive techniques and possibility of fixation of PRA on middle 1/3rd clavicle and utilization of image-guided navigation for anterior cervical spine procedures have created a paradigm shift in surgical accuracy in anterior cervical approaches allowing motion preservation.

## Conflict of interest

The authors declare that they have no conflicts of interest in relation to this article.
